# Bioactive Properties of *Persea americana* Peel Extract and Their Role in Hypercholesterolemia Management and Cardiovascular Health

**DOI:** 10.3390/foods14142482

**Published:** 2025-07-16

**Authors:** Laura M. Teixeira, Catarina P. Reis, Rita Pacheco

**Affiliations:** 1 Departamento de Química e Bioquímica, Faculdade de Ciências, Universidade de Lisboa, 1749-016 Lisboa, Portugal; fc57476@alunos.ciencias.ulisboa.pt; 2 Centro de Química Estrutural, Institute of Molecular Sciences, Faculdade de Ciências, Universidade de Lisboa, 1749-016 Lisboa, Portugal; 3 Institute for Medicines (iMed.ULisboa), Faculdade de Farmácia, Universidade de Lisboa, 1649-003 Lisboa, Portugal; 4 Instituto de Biofísica e Engenharia Biomédica (IBEB), Faculdade de Ciências, Universidade de Lisboa, 1749-016 Lisboa, Portugal; 5 Departamento de Engenharia Química, Instituto Superior de Engenharia de Lisboa, 1959-007 Lisboa, Portugal

**Keywords:** cardiovascular diseases, hypercholesterolemia, *Persea americana*, bioactive compounds, pyridoxine + O-Hex

## Abstract

Cardiovascular diseases remain the leading cause of death worldwide, with hypercholesterolemia being a major contributing risk factor. Although cholesterol-lowering drugs are widely available, concerns about several adverse side effects have increased the demand for natural alternatives, with the most common approaches involving the incorporation of foods rich in bioactive compounds into the diet. To explore this growing interest in food-based strategies for cardiovascular health, this study formulated and evaluated an aqueous peel extract of *Persea americana* to assess its potential role as a complementary approach to managing hypercholesterolemia. The extract was characterized, revealing the presence of various bioactive compounds, including pyridoxine-O-Hex, which was identified for the first time in a *P. americana* extract component. The safety profile of the extract was confirmed through in vivo assessment. Furthermore, the extract demonstrated protective effects against oxidative stress in HepG2 cells. Additionally, permeability studies using Caco-2 cells, as a model of the gastrointestinal barrier, indicated that the extract effectively reduced cholesterol’s permeation. In summary, these findings suggest that *P. americana* peel extract may serve as a promising natural product for functional foods for cardiovascular health and hypercholesterolemia management.

## 1. Introduction

Cardiovascular diseases (CVDs) are considered the leading cause of death globally, accounting for approximately 30% of all deaths in the world. These diseases predominantly affect the elderly, with 86% of cases occurring in individuals over the age of 80 [1,2,3]. Apart from age, several risk factors contribute to the development of CVDs, particularly lifestyle related factors, such as smoking and unhealthy diets rich in saturated fats, which are strongly associated with obesity and hypercholesterolemia. These behaviors can lead to medical conditions, such as hypertension and several metabolic syndromes, further increasing the risk of CVDs [2,3,4].

Among these, hypercholesterolemia, a condition characterized by elevated blood cholesterol levels, is recognized as a major contributing factor in the development of CVDs [5,6]. Dysregulation of cholesterol metabolism is a key factor for hypercholesterolemia. In the human organism, cholesterol can be obtained through two main routes: exogenous cholesterol obtained from the diet and endogenous cholesterol synthesized in the liver [7].

Maintaining cholesterol levels within the recommended parameters is essential for preventing cardiovascular disorders. This can be achieved through healthy lifestyle habits, such as regular physical activity and a balanced diet [4,8]. However, for some individuals, lifestyle changes may not be sufficient to manage cholesterol levels, making the use of cholesterol-lowering medication necessary [8].

Cholesterol-lowering drugs include statins, a key enzyme in the biosynthesis of cholesterol in the liver, which inhibits 3-hydroxy-3-methylglutaryl coenzyme A reductase (HMGR) [9,10]. Another commonly prescribed drug, ezetimibe, inhibits cholesterol absorption in the intestine by blocking the sterol transporter, Niemann-Pick C1-Like 1 (NPC1L1) [11].

While these pharmacological therapies are effective, their chronic use is often associated with adverse side effects, such as muscle pain and/or hepatotoxicity [4]. As a result, there has been a growing interest in complementary approaches, particularly food supplements derived from natural sources, which are viewed as safe, health-promoting, and culturally acceptable alternatives that may help reduce the use of medication [8,12].

Among these, foods and supplements rich in phenolic compounds have been extensively studied due to their wide range of bioactive properties, in addition to their potent antioxidant activity, which protects the organism against oxidative stress, recognized as a major contributor to the development of various diseases, CVDs included [13,14]. Moreover, such supplements represent a promising option for individuals with moderately elevated cholesterol levels, not meeting the criteria for prescription but requiring prevention or milder intervention often seeking natural effective alternatives [15].

Various food supplements have been used to improve cardiovascular health, such as red yeast rice, fishomega-3 fatty acids, and phytosterols [16]. For instance, green tea leaves (*Camellia sinensis*), rich in polyphenolic flavonoids, such as catechins, have shown to reduce plasma total cholesterol levels and improve lipid metabolism [17]. Additionally, extracts from plants such as *Centaurium erythraea* have also demonstrated cholesterol-reducing properties associated with their flavonoid content [18]. Another example is *Vernonia condensata*, a chlorogenic acid, along with other caffeoylquinic acid derivatives containing plants, which demonstrate the capacity to inhibit HMGR, as well as lower cholesterol permeation in an intestinal lining cell model [19].

In addition, plant-based extracts obtained from agriculture by-products, such as peels of *Annona cherimola*, rich in rutin [20], and leaves of *Actinidia deliciosa*, abundant in flavonoids [21], have been used to prepare extracts, that demonstrate potential for hypercholesterolemia management. The bioactive compounds in these by-products were described to reduce cholesterol absorption in the intestinal lining, inhibiting HMGR and enhancing antioxidant defenses, making them promising candidates for inclusion in functional foods for cardiovascular health [22,23]. Furthermore, the use of waste rich in bioactive compounds offers a sustainable strategy that aligns with circular economy principles and supports environmentally friendly approaches.

Supporting this growing trend, the present work focuses on the valorization of high-value compounds contained in the peels of *Persea americana*, commonly known as avocado. The increasing demand for this fruit can be attributed to the growing perception of its numerous health properties, including cardiovascular protection and antioxidant activity [24]. However, the avocado processing industry generates a large volume of by-products, mainly peels and seeds, representing around 13 to 18% of the fruit’s dry weight, which normally end up being discarded [25,26,27]. These by-products have a high content of many bioactive compounds, such as phenolic acids, flavonoids, tannins, and others, many of which have been associated with health benefits [26,27,28]. Therefore, avocado by-products are ideal for the extraction of phenolic and other bioactive compounds with promising potential as functional ingredients for the development of food supplements. Additionally, recycling of these by-products would reduce production costs and minimize the avocado industry’s environmental impact [28,29].

Given this, in this work, an aqueous extract from *P. americana* peel was evaluated regarding its potential role in CVD prevention, particularly in managing hypercholesterolemia. This study focused on its bioactive compound content, antioxidant capacity, and effects on intestinal cholesterol’s permeability.

## 2. Materials and Methods

### 2.1. Materials

#### 2.1.1. Chemicals

Dulbecco’s Modified Eagle Medium (DMEM), Roswell Park Memorial Institute Medium (RPMI-1640), and fetal bovine serum (FBS) were acquired from Biowest (Nuaillé, France). Gallic acid, *tert*-butyl hydroperoxide (TBHP), 2′-7′-dichlorofluoroscin diacetate (DCFDA), cholesterol, the Folin–Ciocalteu reagent, and sodium carbonate were obtained from Sigma-Aldrich (Barcelona, Spain). Antimycotic and L-glutamine were purchased from Corning (New York, NY, USA). D-glucose was acquired from HiMedia Laboratory (Mumbai, India). Calcium chloride, sodium bicarbonate, magnesium sulphate anhydrous, and monopotassium phosphate were purchased from Merck kGaA (Darmstadt, Germany). Disodium hydrogen phosphate and trifluoroacetic acid were obtained from PanReac (Barcelona, Spain). Dimethyl sulfoxide ultrapure (DMSO) were obtained from VWR (Radnor, PA, USA). Magnesium chloride hexahydrate, sodium chloride, and potassium chloride were acquired from Honeywell (Charlotte, NC, USA). Formic acid, acetonitrile (ACN) LC-MS grade Optima, and methanol were purchased from Fisher Scientific (Hampton, VA, USA). All chemicals were of analytical grade.

#### 2.1.2. Cell Line and Cell Culture

Two human cancer cell lines were used in this study, hepatocellular carcinoma cell line (HepG2) (ECACC 85011430) and colon carcinoma cell line (Caco-2) (ECACC 86010202), both obtained from the European Collection of Authenticated Cell Cultures. Caco-2 cells were cultured in RPMI medium, while HepG2 cells were maintained in DMEM. Both media were supplemented with 10% FBS, 100 U/mL penicillin, 100 U/mL streptomycin, and 2 mM L-glutamine. Culture media were refreshed every 42–78 h. The cells were incubated in T75 flasks at 37 °C in a humidified atmosphere containing 5% CO_2_.

### 2.2. Methods

#### 2.2.1. Preparation of *Persea americana* Aqueous Extract

The aqueous extract was prepared by decoction from washed fresh peels of *P. americana* (Hass variety). The peels were boiled in water (100 g/L) for 15 min. The resulting decoction was filtered using Whatman filter paper n° 4, frozen, and subsequently freeze-dried (Heto^®^, PowerDry LL3000, Milford, MA, USA) to obtain a dry extract. The final extract was stored at −20 °C until further use.

#### 2.2.2. Quantification of Phenolic Content

The total phenolic content (TPC) of the extract was determined using the Folin–Ciocalteu method described by Oktay et al. [30]. The absorbance was measured at 760 nm and gallic acid (GA) was used as a standard with a series of concentrations (0–500 μg/mL) to obtain a standard curve (y = 0.0247x − 0.0067; R^2^ = 0.992). The TPC of the extract was determined as milligrams of gallic acid equivalents (GAEs) per gram of dry extract. The assay was carried out in quadruplicate.

#### 2.2.3. UHPLC-ESI-QTOF-MS Analysis of Bioactive Compounds 

The bioactive compounds present in the extract were tentatively identified by ultra-high performance liquid chromatography coupled with a quadrupole time-of-flight mass spectrometer equipped with an electrospray ionization source (UHPLC-ESI-QTOF-MS/MS) (maXis Impact II, Bruker, Bremen, Germany) and an AcquityTM Premier CSH C18 1.7 µm VanguardTM FIT 2.1 × 100 mm column (Waters, Milford, MA, USA). The mobile phase consisted of 0.1% of formic acid in water (solution A) and 0.1% of formic acid in ACN (solution B). The gradient conditions were as follows: 0–7 min 95% A, 5% B; 7–15 min 50% A, 50% B; 15–25 min 30% A, 70% B; 25–35 min 15% A, 85% B; 35–38 min 95% A, 5% B. The flow rate was 0.25 mL/min for the first 7 min, followed by 0.30 mL/min up to 38 min. The extract was injected at a concentration of 1.0 mg/mL in ultrapure water.

The analysis was carried out in the positive ESI mode, with the mass spectrum acquired in a 50–1500 *m*/*z* range, a capillary potential of 4000 V, dry heat at 200 °C, a nebulizer at 2 bars, a dry gas flow of 8.0 L/min, and a collision energy of 10 eV. For the negative ESI mode, the spectrum conditions were similar, with the following exceptions: the capillary potential was set to 3500 V, the dry gas flow was 4.0 L/min, and the collision energy was −5 eV.

The results obtained from mass spectrometry were first processed and analyzed using the Global Natural Products Social Molecular Networking (GNPS) platform [31] and DataAnalysis 4.4 software (Bruker^®^, Bremen, Germany). Compound identification was based on spectral matching with the GNPS library, considering a cosine score threshold of 0.7 or higher to ensure reliable annotation, with a minimum of 6 shared peaks. Additionally, only compounds with a mass ≤ 5 ppm were considered to enhance identification accuracy, and the fragments of ions with a low intensity (<10%) were not considered. Subsequently, in the DataAnalysis 4.4 software (Bruker, Bremen, Germany), only compounds exhibiting a relative intensity above 10^4^ were considered for further analysis to ensure that detected compounds were of sufficient abundance for reliable detection. Based on the Metabolomics Standards Initiative (MSI) guidelines, the identifications fall under Level 2 (putatively annotated compounds), supported by MS^2^ spectral matching and literature data.

#### 2.2.4. Reactive Oxygen Species (ROS) Production

The quantification of reactive oxygen species (ROS) produced in the HepG2 cell line was assessed. HepG2 cells were plated at a concentration of 2.5 × 10^4^ cells/well in a black, transparent-bottom 96-well microplate and incubated for 24 h. Afterward, a solution of the 2′,7′–dichlorodifluorescin diacetate (DCFDA) (0.1 mM) was added to the cells and they were incubated in the dark for 45 min. The solution was then removed, and the following samples were applied: a control with only the PBS, *tert*-butyl hydroperoxide (TBHP) at 200 µM, the extract at 0.164 mg/mL, and a combination of the extract and THBP with their final concentrations being 0.164 mg/mL and 200 µM, respectfully. The microplate was incubated for 6 h in the dark, after which fluorescence was measured with excitation at 495 nm and emission at 527 nm, both at 37 °C (SpectraMax^®^ GEMINI XPS/EM, Molecular Devices^®^, San Jose, CA, USA). ROS production was determined using Equation (1) by considering that the fluorescence produced by the control group was 100%.

(1) $$\;\mathrm{ROS}\;\mathrm{Production}\;\left(\%\right)\;=\frac{{\mathrm{Abs}}_{\mathrm{Sample}}}{{\mathrm{Abs}}_{\mathrm{Control}}}\;\times \;100$$

where Abs_Sample_ corresponds to the absorbance of the samples and Abs_Control_ corresponds to the absorbance of the control. The assays were carried out in quadruplicate.

#### 2.2.5. In Vivo Safety Assay

For the in vivo safety assay, *Artemia salina* was used as a model following the protocol described by Lopes et al. [32]. Dehydrated eggs of *A. salina* (JBL Artemio Pur^®^ GmBh & Co., Neuhofen, Germany) were hatched and then incubated with the extract at 0.240 mg/mL for 24 h. The positive and negative controls were DMSO and artificial sea water, respectfully. The mortality rate (%) of *A. salina* was determined using Equation (2) and the assays were carried out in quadruplicate.

(2) $$\;\mathrm{Mortality}\;\mathrm{Rate}\;\left(\%\right)\;=\frac{{\mathrm{Dead}}_{A.\;\mathrm{salina}}}{{\mathrm{Total}}_{A.\;\mathrm{salina}}}\;\times \;100$$

#### 2.2.6. Assessment of Cholesterol’s Permeability in a Gastrointestinal Model

The cholesterol permeation assays were carried out by following the protocol described by Arantes et al. [19]. Caco-2 cells were cultured in a 12-well microplate with inserts (A = 1.1 cm^2^) and maintained until differentiation. To evaluate the integrity of the membrane, transepithelial electrical resistance (TEER) of the formed membrane was measured. When the TEER value exceeded 250 Ω·cm^2^, it was considered that the membrane was formed.

Subsequently, 0.5 mL of the solutions to be analyzed was added on the apical side of each insert: cholesterol (5 mM) (control), extract (0.240 mg/mL), extract (0.240 mg/mL) with cholesterol (5 mM), and ezetimibe (0.1 mM) with cholesterol (5 mM). On the basolateral side, 1.5 mL of the Hank’s Balanced Salt Solution (HBSS) was added. The cells were incubated at 37 °C in a 5% CO_2_ atmosphere for 6 h. After this period, the basolateral solution of each well was collected and analyzed by high-performance liquid chromatography coupled to a diode-array detector (HPLC-DAD) using an Elite LaChrom^®^ (HITACHI, VWR, Tokyo, Japan), with an Autosampler L-2200, a Column Oven L-2300, and a Diode Array Detector L 2455 (VWR, Radnor, PA, USA). The column used was LiChroCART^®^ 250-4 LiCrosphere^®^100 RP-8 (5 μm) (Merk, Darmstadt, Germany). The conditions followed were the ones presented by Pinto et al. [4].

The percentage of cholesterol permeated was determined using Equation (3), where the cholesterol permeated after 6 h for the control group was considered to represent 100% permeation.

(3) $$\;\mathrm{Cholesterol}\;\mathrm{Reduction}\;(\%)\;=\frac{{\mathrm{Cholesterol}}_{\mathrm{Sample}}}{{\mathrm{Cholesterol}}_{\mathrm{Control}}}\;\times \;100$$

where Cholesterol_sample_ refers to the cholesterol on the basolateral side of the cells after 6 h of incubation of each sample, and Cholesterol_control_ refers to the cholesterol on the basolateral side of the cells treated only with cholesterol after 6 h. The assays were carried out in duplicate.

#### 2.2.7. Permeation of the Extract Compounds

The permeation of the extract in Caco-2 cells was evaluated for three phenolic compounds previously identified and quantified [33], namely chlorogenic acid, catechin, and epicatechin. For this purpose, the protocol described in Section 2.2.6 was followed, where two samples were tested on the apical side: the extract (0.240 mg/mL) and the extract (0.240 mg/mL) with cholesterol (5 mM). After 6 h, a sample was collected from the basolateral side and analyzed by HPLC-DAD, using a LiChroCART^®^ 250-4 LiChrospher^®^ 100 RP-18 (5 µm) column (Merck KGaA, Darmstadt, Germany), following the conditions described in a previous study [33]. The apparent permeability coefficient (P_app_) was determined using Equation (4).

(4) $$\;P_{app}=\frac{V}{A\times C_{i}}\times \frac{C_{f}}{t}$$

where V is the volume of the apical side in mL, A is the area of the insert membrane in cm^2^ (1.1 cm^2^), C_i_ is the initial concentration on the apical side in mg/mL, C_f_ is the concentration on the basolateral side in mg/mL, and t is the time in seconds.

#### 2.2.8. Statistical Analysis

The results are presented as mean ± standard deviation. Additionally, statistical analysis of variance was assessed using ANOVA (Microsoft Office 365, Washington, DC, USA), with a *p* = 0.05.

## 3. Results

In this study, an aqueous peel extract of *P. americana* was formulated and evaluated for its potential application in hypercholesterolemia management. The extract was prepared using water, thereby making the extraction process environmentally friendly by eliminating the use of organic solvents and reducing the risk of toxicity.

### 3.1. Quantification of Extract Total Phenolic Content

The total phenolic content (TPC) of the extract was determined using the Folin–Ciocalteu method, using gallic acid (GA) as a standard. The TPC obtained for the extract was 159.07 ± 0.02 mg GAE/g of dry extract, confirming the presence of phenolic compounds. When comparing this TPC value with those reported in previous studies from avocado peel extracts [26,34], the results fall within the expected range. However, variations in TPC values are common in natural product preparations due to differences in extraction methods, such as gridding or sonication of the extracts [35,36], the maturity state of the fruit [37], and storage conditions [38]. These factors may influence the overall yield and composition of the bioactive compounds present in the extract.

### 3.2. Identification of Bioactive Compounds by UHPLC-ESI-QTOF-MS

The bioactive compounds present in the avocado peel extract were identified by ultra-high performance liquid chromatography coupled to a quadrupole time-of-flight mass spectrometer equipped with an electrospray ionization source (UHPLC-ESI-QTOF-MS/MS). The chromatograms obtained, both in negative and positive ESI mode, are presented in Supplementary Material Figure S1.

A total of 18 compounds were tentatively identified, as detailed in Table 1 (negative ionization mode) and in Table 2 (positive ionization mode). These included a variety of bioactive compounds such as phenolic acids, flavonoids, vitamins, amino acids, fatty acids, and others. Notably, phenolic acids such as mucic acid (**1**), quinic acid (**2**), chlorogenic acid (**3**), and caffeic acid (**14**), as well as flavonoids like (+)-catechin (**13**) and (−)-epicatechin (**15**), were among the compounds identified. In addition, pyridoxine + O-Hex (**6**) (Figure S2) was identified for the first time in a *P. americana* extract. Although this compound had previously been reported in *Pisum sativum* (pea) [39], its detection in *P. americana* represents a novel finding.

### 3.3. Reactive Oxygen Species (ROS) Protection in Hepatic Cell Line HepG2

Building upon preliminary results that indicate strong antioxidant activity (AA) of the aqueous *P. americana* peel extract, yielding an EC_50_ value of 6.0 ± 0.2 µg/mL by DPPH assay [33], we aimed to evaluate this extract’s ability to protect liver cells from oxidative stress. HepG2 cells, a human hepatocellular carcinoma cell line, were used as a model due to the liver’s central role in lipid metabolism, xenobiotic metabolism, and antioxidant defense. This makes it a relevant system for assessing the biological effects of food-derived compounds, particularly those targeting hypercholesterolemia and oxidative stress.

To investigate this, HepG2 cells were exposed to *tert*-butyl hydroperoxide (TBHP; 200 µM) to induce oxidative damage. A parallel group was treated with a combination of TBHP (200 µM) and the extract at a final concentration of 0.164 mg/mL, the IC_50_ concentration previously determined for this cell line [33], to evaluate the extract’s potential cytoprotective effects. A negative control group, consisting of HepG2 cells incubated with PBS only, was included to serve as a baseline for comparison.

**Figure 1:**
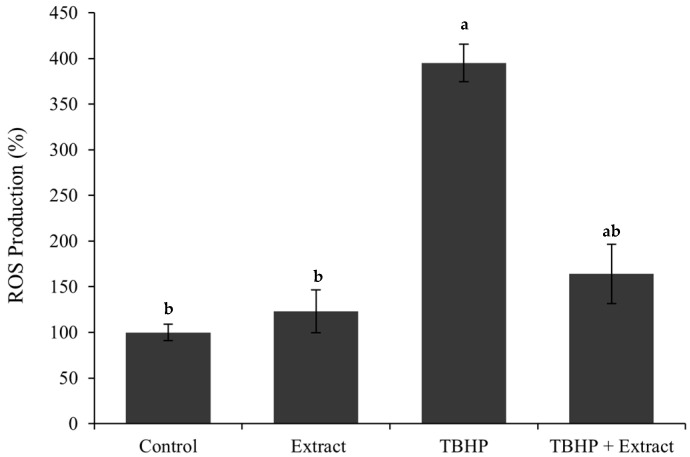
Measurement of ROS production (% of control) after 6 h incubation. Where “a” corresponds to samples that are statistically different from control and “b” corresponds to samples that are statistically different from TBHP, according to a one-way ANOVA (*p* ≤ 0.05).

The results, presented in Figure 1, show as expected that exposure to TBHP alone induced a significant increase in intracellular ROS production, reaching approximately 395% relative to baseline levels, indicating a high level of oxidative stress. In contrast, co-treatment with TBHP and the extract (TBHP + extract) resulted in a substantial reduction in ROS production levels, decreasing to 164%. This demonstrates the extract’s effective antioxidant capacity and its capacity to protect against ROS-inducing agents.

### 3.4. In Vivo Safety Assays

**Figure 2:**
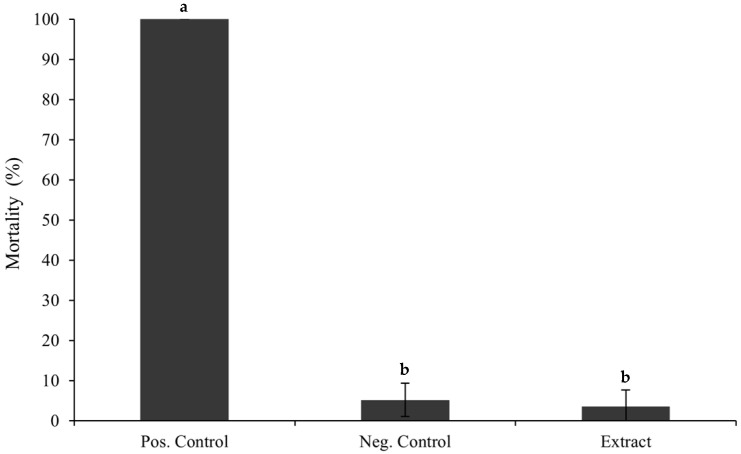
The mortality rate of *A. salina*, 24 h after being incubated with the extract (0.240 mg/mL). The positive and negative controls were 100% of DMSO and artificial sea water, respectively. Where “a” corresponds to the samples that are statistically different from the negative control and “b” corresponds to the samples that are statistically different from the positive control, according to a one-way ANOVA (*p* ≤ 0.05).

To extend our investigation and complement previous in vitro safety data of this extract [33], an in vivo assay was performed. The *Artemia salina* (brine shrimp) lethality assay was employed as an additional model for toxicity screening. The extract was tested at a concentration of 0.240 mg/mL, corresponding to the IC_50_ value previously determined in the Caco-2 cell line [33]. As shown in Figure 2, exposure to the extract resulted in a mortality rate of *A. salina* below 5%, indicating low toxicity.

### 3.5. Anti-Hypercholesterolemic Effect—Reduction in Cholesterol’s Permeability

Given the composition profile of the extract (Table 1 and Table 2), which includes a variety of bioactive compounds, its potential role in reducing hypercholesterolemia was of particular interest. Compounds such as flavonoids like (+)-catechin (**13**) and (−)-epicatechin (**15**) were reported to possess strong antioxidant properties, which help to mitigate oxidative stress, a key factor in the development of atherosclerosis and other CVDs [17,40]. Moreover, chlorogenic acid (**3**) has been shown to possess lipid-lowering effects, including the ability to reduce cholesterol absorption and modulate lipid metabolism pathways [19,41]. In addition to the isolated compounds, as previously mentioned, plant extracts rich in these molecules have also demonstrated similar biological activities, further supporting their relevance in cardiovascular health [17,19].

In this study, human Caco-2 cell line, differentiated into a monolayer, was used as an in vitro model of the intestinal epithelial barrier. These cells are widely recognized for their ability to differentiate into a functional and morphological state resembling human enterocytes, making them a well-established system for studying intestinal absorption and transport processes [42]. This model allowed us to investigate whether the extract could influence cholesterol permeation, a key factor for managing blood cholesterol levels.

For this purpose, the cells were treated with the extract at a concentration of 0.240 mg/mL, corresponding to the IC_50_ value previously determined in Caco-2 cell cytotoxicity assays [33]. To simulate dietary cholesterol intake, a 5 mM cholesterol solution was applied on the apical side of the monolayer. The percentage of reduction in cholesterol permeability was calculated by comparison to a control condition, where the monolayer was exposed only to the cholesterol solution, which represents 100% permeability.

**Figure 3:**
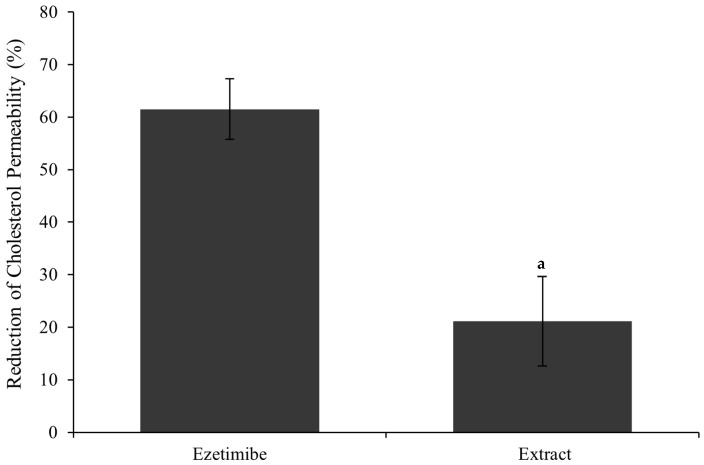
Cholesterol permeation reduction in a Caco-2 model for ezetimibe (0.01 mM) and extract (0.240 mg/mL). Cholesterol solution (5 mM) in basolateral was considered 100% permeation. Where “a” correspond to sample that is statistically different from ezetimibe, according to one-way ANOVA (*p* ≤ 0.05).

As shown in Figure 3, treatment with the extract resulted in a significant reduction in cholesterol permeability by 22 ± 6%, suggesting that the extract may effectively limit cholesterol intestinal absorption. When compared with ezetimibe, a clinically approved lipid-lowering drug that was shown to reduce cholesterol permeation by 62 ± 5%, the extract performance is noteworthy, considering that the extract is a mixture of bioactive compounds and not a pure drug.

### 3.6. Permeability of Chlorogenic Acid, Catechin, and Epicatechin: The Extract’s Compounds

To further elucidate the mechanisms and effects of the extract, the permeability across the Caco-2 monolayer of the individual bioactive compounds, chlorogenic acid (**3**), catechin (**13**), and epicatechin (**15**), the three major compounds previously identified in the extract [33], were evaluated. This approach aimed not only to determine which specific constituents could influence intestinal cholesterol absorption, but also to assess their transport properties, providing insights into their bioavailability and potential to exert effects in other parts of the body following consumption. A common limitation in the oral administration of dietary supplements is the intestinal absorption of bioactive compounds, which can affect their effects when consumed.

To address this, the permeation of chlorogenic acid (**3**), catechin (**13**), and epicatechin (**15**) was assessed by determining their apparent permeability coefficient (P_app_) using the Caco-2 cell model. The extract was applied alone and in combination with cholesterol to the apical side of the monolayer of the cells. The P_app_ coefficient serves as a key parameter to evaluate the permeability of the compound across the intestinal membrane. A P_app_ value lower than 1.0 × 10^−6^ cm/s indicates low permeability, while a P_app_ value greater than 10.0 × 10^−6^ cm/s suggests high permeability. Compounds with values between these two thresholds are classified as having moderate permeability. Additionally, a P_app_ value higher than 2.8 × 10^−7^ cm/s indicates that the permeation of the compounds is likely facilitated through membrane transport [43].

Based on the results shown in Table 3, chlorogenic acid (**3**) exhibited moderate permeability, while both catechin (**13**) and epicatechin (**15**) demonstrated high permeability. The moderate permeability of chlorogenic acid (**3**) is consistent with previous studies, indicating that this compound typically has low to moderate intestinal absorption efficiency [44]. Catechin (**13**) demonstrated high permeability, which aligns with findings from another work showing that catechins are highly bioavailable and readily absorbed in the gastrointestinal tract. Similarly, epicatechin (**15**) exhibited high permeability, as reported in the same study, in which epicatechin was found to have either moderate or high intestinal absorption [45]. The obtained permeability values suggest that the bioactive compounds of the extract can effectively cross the intestinal epithelium, which is a crucial step for their absorption and systemic distribution.

It was additionally seen that, when the extract was tested in combination with cholesterol, only epicatechin (**15**) maintained its permeability across the Caco-2 monolayers, suggesting that chlorogenic acid (**3**) and catechin (**13**) may interfere with cholesterol permeation.

## 4. Discussion

Over the years the demand for plant-based supplements has been growing significantly, driven by consumer preference for natural products, which are viewed as a safer and healthier complement or alternative to conventional therapies [12]. Specifically, in the treatment of hypercholesterolemia, where the current drugs available on the market are frequently associated with some side effects, plant-based supplements are being researched [8]. In response to these concerns, numerous studies have focused on evaluating plant-based extracts and their bioactive compounds as potential alternatives for managing hypercholesterolemia by exploring their mechanisms of action [4,46,47]. This shift towards natural remedies is also based on the longstanding knowledge in traditional medicine across many cultures, where plant-based supplements, such as functional foods and dietary supplements, have been used for various therapeutic purposes.

This work evaluates the potential of a peel extract of *P. americana*, obtained through an environmentally friendly extraction method aligned with circular economy principles, as a novel complementary strategy for the prevention and management of CVDs, with a specific focus on targeting hypercholesterolemia.

Phytochemical profiling by UHPLC-QTOF-MS/MS revealed a diverse array of bioactive compounds in the *P. americana* peel extract, including flavonoids, such as catechin (**13**) and epicatechin (**15**), phenolic acids, such as chlorogenic acid (**3**) and caffeic acid (**14**), and other bioactive compounds, namely amino acids and a vitamin B6 derivative, such as pyridoxine + O-Hex (**6**), with the latter identified for the first time in *P. americana*. Most of these compounds have been previously reported in *P. americana* and are associated with various health benefits, including cardioprotective properties [24,48,49,50,51]. For instance, amino acids such as L-glutamine (**7**) have been linked to reduced CVD mortality [52]. Another compound, glutathione (**10**), is known for its antioxidant effect and protection from oxidative stress [24]. Pyridoxine + O-Hex (**6**), a vitamin B6 derivative, may also contribute to the extract’s health-promoting effects, as pyridoxine, also known as vitamin B6, has been associated with reducing the risk factor for CVDs [53]. Phenolic compounds, particularly chlorogenic acid and catechins, have been extensively studied and reported for health and well-being potential and are widely documented for their cardioprotective properties [54], including antioxidant and lipid-lowering potential [17,19,55].

Given the relevance of oxidative stress in the development and progression of CVDs, the presence of antioxidant-rich compounds in the extract and the exhibited capacity to protect HepG2 liver cells from oxidative stress, by a reduction in intracellular ROS production, reinforces the potential of avocado-based extracts for the prevention and management of oxidative stress-associated diseases. Similar extracts derived from avocado peels and seeds have been shown to reduce ROS-induced stress in other cell line models, such as Caco-2 cells [56], further supporting this idea.

The safety profile of the extract, indicated by the low mortality rate in the *Artemia salina* lethality assay, aligns with previous cytotoxicity data in human cell lines [33], suggesting the extract’s biocompatibility and minimal risks upon exposure. Additionally, this low toxicity also indicates a reduced environmental impact, especially when compared to other pharmaceutical and supplements residues, which often pose ecotoxic risks and exhibit environmental burden. These findings also suggest that the extract holds significant potential for further development and application, particularly as a functional food ingredient, where both environmental sustainability and consumer safety are critical factors to consider.

The cholesterol permeability assays performed revealed that the extract reduced cholesterol permeation in a human model of the intestinal lining, highlighting its potential as a natural alternative or complementary strategy to modulate cholesterol homeostasis for the prevention and management of hypercholesterolemia and CVDs. Supporting this potential, other studies have shown that the administration of avocado extracts to rats resulted in a significant reduction in cholesterol blood levels [57,58,59]. Given the extract complex and unrefined nature when compared to single-target drugs like ezetimibe, these results seem noteworthy, as unlike conventional drugs, the extract’s multitarget activity could offer synergistic benefits through various pathways, as demonstrated by the exhibited antioxidant activity and oxidative stress protective effects. The ability of the extract’s bioactive compounds to cross the intestinal barrier makes them systemically available and potentially reaching organs such as the liver, where they may exert additional effects. As demonstrated in this study, the extract significantly mitigated cellular oxidative stress, indicating hepatic protective potential. Given the potential of the extract to reach the liver, it would be interesting to further investigate its protective effect but also the inhibitory effect on liver enzymes, such as HMGR, and to explore additional mechanisms underlying its cholesterol-lowering effects.

Interestingly, in the presence of cholesterol, only epicatechin successfully permeated the membrane, suggesting that chlorogenic acid and catechin can interfere with cholesterol permeation, potentially by interacting with cholesterol or modulating membrane transporter proteins involved in lipid absorption. This dual function, blocking cholesterol while allowing selective compound absorption, could also be a valuable trait for functional food development.

Taken together, these findings support the notion that avocado-based supplements may provide a natural and effective complement for managing hypercholesterolemia.

## 5. Conclusions

In conclusion, this study highlights the promising potential of an aqueous *P. americana* peel extract as a functional food for CVD prevention. The extract’s bioactive compounds contribute to reducing oxidative stress and cholesterol permeability. These effects, together with the low toxicity profile and potential for the systemic bioavailability of its constituents, suggest a multifaceted mode of action that could benefit cardiovascular health, particularly in a multifactorial disease such as hypercholesterolemia.

However, while these results are promising, further studies should focus on the effect of the extract’s mechanisms, along with animal studies to confirm the extract’s efficacy and explore its integration into daily dietary practices. Ultimately, *P. americana* peel extract could offer a natural and accessible strategy to complement current CVD prevention methods and contribute to tackling a major threat in world health.

## Figures and Tables

**Table 1:** Tentative identification of the compounds in the peel extract of *Persea americana*, detected by UHPLC-ESI-QTOF-MS/MS in negative ionization mode.

Rt (min)	[M − H]^−^ *m*/*z*	Molecular Formula	Error (ppm)	MS2 Fragmentation	Name	N°
1.9	209.0668	C_6_H_10_O_8_	−0.52	71.0141 (41.20%); 59.0147 (100.0%)	Mucic Acid	1
2.2	191.0563	C_7_H_12_O_6_	−3.08	93.0345 (26.1%); 87.0088 (16.3%); 85.0294 (100.0%); 59.0143 (39.8%)	Quinic Acid	2
2.4	353.0879	C_16_H_18_O_9_	8.80	192.0588 (12.5%); 191.0561 (100.0%); 93.0347 (15.6%); 85.0295 (30.6%)	Chlorogenic Acid	3
2.5	191.0197	C_6_H_8_O_7_	0.14	111.0081 (17.2%); 87.0787 (69.3%); 85.0294 (100.0%); 67.0195 (27.0%); 57.0351 (37.2%)	Citric Acid	4

**Table 2:** Tentative identification of the compounds in the peel extract of *Persea americana*, detected by UHPLC-ESI-QTOF-MS/MS in positive ionization mode.

Rt (min)	[M + H]^+^ *m*/*z*	Molecular Formula	Error (ppm)	MS2 Fragmentation	Name	N°
1.5	105.1154	C_5_H_14_NO^+^	0.86	104.1067 (52.2%); 60.0787 (100.0%); 58.0631 (30.6%)	Choline Cation	5
1.7	332.1348	C_14_H_21_NO_8_	−2.43	332.1357 (76.3%); 314.1252 (52.8%); 152.0686 (42.0%); 124.0752 (41.4%); 108.0805 (100.0%)	Pyridoxine + O-Hex	6
1.8	147.0759	C_5_H_10_N_2_O_3_	4.89	130.0493 (13.4%); 85.0472 (10.8%); 84.0432 (100.0%); 56.0472 (20.5%)	L-Glutamine	7
1.9	213.0971	C_7_H_16_O_7_	−1.04	123.0439 (41.4%); 111.0432 (51.9%); 99.0433 (49.9%); 85.0276 (57.6%); 81.0326 (76.7%); 69.0318 (100.0%)	Perseitol	8
2.2	182.0806	C_9_H_11_NO_3_	3.13	136.0748 (100.0%); 123.0451 (71.4%); 119.0491 (61.2%); 95.0504 (30.9%); 91.0543 (97.5%);	L-Tyrosine	9
2.2	308.0916	C_10_H_17_N_3_O_6_S	−1.44	305.1569 (100.0%); 130.0499 (36.4%); 84.0429 (42.2%);	Glutathione	10
2.2	268.1043	C_10_H_13_N_5_O_4_	1.01	136.0612 (100.0%)	Adenosine	11
2.4	579.1493	C_30_H_26_O_12_	0.70	579.1502 (15.8%); 287.0547 (26.2%); 271.0630 (24.7%); 127.0385 (100.0%); 123.0434 (44.2%)	Procyanidin B2	12
2.5	291.0869	C_15_H_14_O_6_	1.76	207.0654 (20.4%); 165.0540 (13.9%); 147.0441 (22.3%); 139.0387 (100.0%); 123.0440 (87.2%)	(+)-Catechin	13
2.5	355.1026	C_16_H_18_O_9_	−0.68	163.0384 (100.0%); 135.0433 (21.8%); 117.0330 (14.3%); 89.0594 (25.8%)	Chlorogenic Acid	3
2.6	181.0501	C_9_ H_8_O_4_	2.89	163.0368 (15.7%); 145.0284 (17.2%); 135.0428 (100.0%); 117.0332 (63.6%); 89.0382 (74.9%)	Caffeic Acid	14
7.1	291.0864	C_15_H_14_O_6_	−2.70	147.0417 (15.3%); 139.0387 (100.0%); 123.0435 (88.1%)	(−)-Epicatechin	15
21.9	256.2641	C_16_H_33_NO	−5.11	256.2640 (100.0%); 88.0745 (20.2%)	Palmitamide	16
22.8	282.2796	C_18_H_35_NO	2.26	282.2790 (21.0%); 265.2537 (10.3%) 247.2424 (15.8%) 97.1054 (46.3%); 83.0844 (81.2%); 69.0683 (100.0%); 57.0679 (67.7%); 55.0522 (67.0%)	Oleamide	17
26.5	284.2958	C_18_H_37_NO	−3.55	285.2999 (31.2%); 284.2529 (100.0%)	Octadecanamide	18

**Table 3:** The apparent permeability coefficient (P_app_) of the three major phenolic compounds from *P. americana* peel extract in the Caco-2 cell model, with and without cholesterol on the apical side. N.D.: Not detected.

Apical Sample	Compound	P_app_ (10^−6^ cm/s)
Extract	Chlorogenic Acid (**3**)	6.5
	Catechin (**13**)	14.4
	Epicatechin (**15**)	24.0
Extract + Cholesterol	Chlorogenic Acid (**3**)	N.D.
	Catechin (**13**)	N.D.
	Epicatechin (**15**)	11.0

## Data Availability

The original contributions presented in this study are included in the article/Supplementary Material. Further inquiries can be directed to the corresponding author(s).

## Supplementary Materials

The following supporting information can be downloaded at: https://www.mdpi.com/article/10.3390/foods14142482/s1. Figure S1: Chromatogram of extract obtained through UHPLC-ESI-QTOF-MS/MS in negative (**a**) and positive (**b**) ESI modes. Figure S2: HRESI-MS2 spectrum data for pyridoxine+ O-Hex (**6**).

## Abbreviations

The following abbreviations are used in this manuscript:

CVD	Cardiovascular Disease
GA	Gallic Acid
GAE	Gallic Acid Equivalents
HMGR	3-hydroxy-3-methylglutaryl coenzyme A reductase
HPLC-DAD	High Performance Liquid Chromatography Coupled to a Diode-Array Detector
NPC1L1	Niemann-Pick C1-Like 1
NPs	Nanoparticles
TPC	Total Phenolic Content
P_app_	Apparent Permeability Coefficient
ROS	Reactive Oxygen Species
TBHP	*tert*-butyl hydroperoxide
UHPLC-ESI-QTOF-MS/MS	Ultra-High Performance Liquid Chromatography coupled to a Quadrupole Time-of-Flight Mass Spectrometer equipped with an Electrospray Ionization Source

## References

[1] Rodgers J.L., Jones J., Bolleddu S.I., Vanthenapalli S., Rodgers L.E., Shah K., Karia K., Panguluri S.K. (2019). Cardiovascular Risks Associated with Gender and Aging. J. Cardiovasc. Dev. Dis..

[2] Kamstrup P.R. (2021). Lipoprotein(a) and Cardiovascular Disease. Clin. Chem..

[3] Casas R., Castro-Barquero S., Estruch R., Sacanella E. (2018). Nutrition and Cardiovascular Health. Int. J. Mol. Sci..

[4] Pinto S., Gaspar M.M., Ascensão L., Faísca P., Reis C.P., Pacheco R. (2022). Nanoformulation of Seaweed Eisenia Bicyclis in Albumin Nanoparticles Targeting Cardiovascular Diseases: In Vitro and In Vivo Evaluation. Mar. Drugs.

[5] Martinez-Hervas S., Ascaso J.F. (2018). Hypercholesterolemia. Encyclopedia of Endocrine Diseases.

[6] Coelho M., Pacheco R. (2023). Anti-Hypercholesterolemia Effects of Edible Seaweed Extracts and Metabolomic Changes in Hep-G2 and Caco-2 Cell Lines. Life.

[7] Patel K.K., Kashfi K. (2022). Lipoproteins and Cancer: The Role of HDL-C, LDL-C, and Cholesterol-Lowering Drugs. Biochem. Pharmacol..

[8] André R., Pacheco R., Bourbon M., Serralheiro M.L. (2021). Brown Algae Potential as a Functional Food against Hypercholesterolemia: Review. Foods.

[9] Camerino G.M., Musumeci O., Conte E., Musaraj K., Fonzino A., Barca E., Marino M., Rodolico C., Tricarico D., Camerino C. (2017). Risk of Myopathy in Patients in Therapy with Statins: Identification of Biological Markers in a Pilot Study. Front. Pharmacol..

[10] Kim S.W., Kang H.J., Jhon M., Kim J.W., Lee J.Y., Walker A.J., Agustini B., Kim J.M., Berk M. (2019). Statins and Inflammation: New Therapeutic Opportunities in Psychiatry. Front. Psychiatry.

[11] Gu J., Zhu N., Li H.F., Zhang C.J., Gong Y.Z., Liao D.F., Qin L. (2022). Ezetimibe and Cancer: Is There a Connection?. Front. Pharmacol..

[12] Ramirez L.I., Kanwugu O.N., Ivantsova M.N. (2022). Impact of Herbal Supplements Nowadays: An Overview. Chim. Techno Acta.

[13] Vilas-Boas A.A., Pintado M., Oliveira A.L.S. (2021). Natural Bioactive Compounds from Food Waste: Toxicity and Safety Concerns. Foods.

[14] Chun O., Balz F., Gardner C., Alekel L., Killen J. Antioxidants: In Depth. https://www.nccih.nih.gov/health/antioxidant-supplements-what-you-need-to-know.

[15] Chen Z.Y., Ma K.Y., Liang Y., Peng C., Zuo Y. (2011). Role and Classification of Cholesterol-Lowering Functional Foods. J. Funct. Foods.

[16] Poli A., Barbagallo C.M., Cicero A.F.G., Corsini A., Manzato E., Trimarco B., Bernini F., Visioli F., Bianchi A., Canzone G. (2018). Nutraceuticals and Functional Foods for the Control of Plasma Cholesterol Levels. An Intersociety Position Paper. Pharmacol. Res..

[17] Luo X.Y., Li N.N., Liang Y.R. (2013). Effects of Ilex Latifolia and Camellia Sinensis on Cholesterol and Circulating Immune Complexes in Rats Fed with a High-Cholesterol Diet. Phytother. Res..

[18] Guedes L., Reis P.B.P.S., Machuqueiro M., Ressaissi A., Pacheco R., Serralheiro M.L. (2019). Bioactivities of Centaurium Erythraea (Gentianaceae) Decoctions: Antioxidant Activity, Enzyme Inhibition and Docking Studies. Molecules.

[19] Arantes A.A., Falé P.L., Costa L.C.B., Pacheco R., Ascensão L., Serralheiro M.L. (2016). Inhibition of HMG-CoA Reductase Activity and Cholesterol Permeation through Caco-2 Cells by Caffeoylquinic Acids from Vernonia Condensata Leaves. Rev. Bras. Farmacogn..

[20] Falé P.L., Ferreira C., Maruzzella F., Helena Florêncio M., Frazão F.N., Serralheiro M.L.M. (2013). Evaluation of Cholesterol Absorption and Biosynthesis by Decoctions of Annona Cherimola Leaves. J. Ethnopharmacol..

[21] Henriques J., Falé P.L., Pacheco R., Florêncio M.H., Serralheiro M.L. (2018). Phenolic Compounds from Actinidia Deliciosa Leaves: Caco-2 Permeability, Enzyme Inhibitory Activity and Cell Protein Profile Studies. J. King Saud. Univ. Sci..

[22] Boggia R., Zunin P., Turrini F. (2020). Functional Foods and Food Supplements. Appl. Sci..

[23] Mihaylova D., Dimitrova-Dimova M., Popova A. (2024). Dietary Phenolic Compounds-Wellbeing and Perspective Applications. Int. J. Mol. Sci..

[24] Bhuyan D.J., Alsherbiny M.A., Perera S., Low M., Basu A., Devi O.A., Barooah M.S., Li C.G., Papoutsis K. (2019). The Odyssey of Bioactive Compounds in Avocado (Persea Americana) and Their Health Benefits. Antioxidants.

[25] Wang W., Bostic T.R., Gu L. (2010). Antioxidant Capacities, Procyanidins and Pigments in Avocados of Different Strains and Cultivars. Food Chem..

[26] Tremocoldi M.A., Rosalen P.L., Franchin M., Massarioli A.P., Denny C., Daiuto É.R., Paschoal J.A.R., Melo P.S., De Alencar S.M. (2018). Exploration of Avocado By-Products as Natural Sources of Bioactive Compounds. PLoS ONE.

[27] de OLIVEIRA C.S., Andrade J.K.S., Rajan M., Narain N. (2022). Influence of the Phytochemical Profile on the Peel, Seed and Pulp of Margarida, Breda and Geada Varieties of Avocado (Persea Americana Mill) Associated with Their Antioxidant Potential. Food Sci. Technol..

[28] Figueroa J.G., Borrás-Linares I., Lozano-Sánchez J., Segura-Carretero A. (2018). Comprehensive Identification of Bioactive Compounds of Avocado Peel by Liquid Chromatography Coupled to Ultra-High-Definition Accurate-Mass Q-TOF. Food Chem..

[29] Rodríguez-Carpena J.G., Morcuende D., Andrade M.J., Kylli P., Estevez M. (2011). Avocado (Persea Americana Mill.) Phenolics, in Vitro Antioxidant and Antimicrobial Activities, and Inhibition of Lipid and Protein Oxidation in Porcine Patties. J. Agric. Food Chem..

[30] Oktay M., Gülçin I., Küfrevioǧlu Ö.I. (2003). Determination of in Vitro Antioxidant Activity of Fennel (Foeniculum Vulgare) Seed Extracts. LWT.

[31] Wang M., Carver J.J., Phelan V.V., Sanchez L.M., Garg N., Peng Y., Nguyen D.D., Watrous J., Kapono C.A., Luzzatto-Knaan T. (2016). Sharing and Community Curation of Mass Spectrometry Data with Global Natural Products Social Molecular Networking. Nat. Biotechnol..

[32] Lopes J., Ferreira-Gonçalves T., Ascensão L., Viana A.S., Carvalho L., Catarino J., Faísca P., Oliva A., de Barros D.P.C., Rodrigues C.M.P. (2023). Safety of Gold Nanoparticles: From In Vitro to In Vivo Testing Array Checklist. Pharmaceutics.

[33] Teixeira L.M., Reis C.P., Pacheco R. (2024). Potential of Application of Natural Product Nanoparticles in Hypercholesterolemia. Chem. Proc..

[34] Ferreira S.M., Santos L. (2022). From By-Product to Functional Ingredient: Incorporation of Avocado Peel Extract as an Antioxidant and Antibacterial Agent. Innov. Food Sci. Emerg. Technol..

[35] Restrepo-Serna D.L., Cardona-Alzate C.A. (2024). The Avocado Peel as a Source of Catechins: A Comparison between Extraction Technologies and the Influence of Fruit Variety. Sustain. Chem. Pharm..

[36] Martínez-Gutiérrez E. (2023). Study of Influence of Extraction Method on the Recovery Bioactive Compounds from Peel Avocado. Molecules.

[37] Mahmood T., Anwar F., Abbas M., Saari N. (2012). Effect of Maturity on Phenolics (Phenolic Acids and Flavonoids) Profile of Strawberry Cultivars and Mulberry Species from Pakistan. Int. J. Mol. Sci..

[38] Klimczak I., Małecka M., Szlachta M., Gliszczyńska-Świgło A. (2007). Effect of Storage on the Content of Polyphenols, Vitamin C and the Antioxidant Activity of Orange Juices. J. Food Compos. Anal..

[39] Tadera K., Mori E., Yagi F., Kobayashi A., Imada K., Imabeppu M. (1985). Isolation and Structure of a Minor Metabolite of Pyridoxine in Seedlings of *Pisum sativum* L.. J. Nutr. Sci. Vitaminol..

[40] Simos Y.V., Verginadis I.I., Toliopoulos I.K., Velalopoulou A.P., Karagounis I.V., Karkabounas S.C., Evangelou A.M. (2012). Effects of Catechin and Epicatechin on Superoxide Dismutase and Glutathione Peroxidase Activity, in Vivo. Redox Rep..

[41] Yan Y., Li Q., Shen L., Guo K., Zhou X. (2022). Chlorogenic Acid Improves Glucose Tolerance, Lipid Metabolism, Inflammation and Microbiota Composition in Diabetic Db/Db Mice. Front. Endocrinol..

[42] Maares M., Haase H. (2020). A Guide to Human Zinc Absorption: General Overview and Recent Advances of in Vitro Intestinal Models. Nutrients.

[43] Ozeki K., Kato M., Sakurai Y., Ishigai M., Kudo T., Ito K. (2015). Evaluation of the Appropriate Time Range for Estimating the Apparent Permeability Coefficient (Papp) in a Transcellular Transport Study. Int. J. Pharm..

[44] Mortelé O., Jorissen J., Spacova I., Lebeer S., Van Nuijs A.L.N., Hermans N. (2021). Demonstrating the Involvement of an Active Efflux Mechanism in the Intestinal Absorption of Chlorogenic Acid and Quinic Acid Using a Caco-2 Bidirectional Permeability Assay. Food Funct..

[45] Rojo-Poveda O., Barbosa-Pereira L., Khattabi C.E., Youl E.N.H., Bertolino M., Delporte C., Pochet S., Stévigny C. (2020). Polyphenolic and Methylxanthine Bioaccessibility of Cocoa Bean Shell Functional Biscuits: Metabolomics Approach and Intestinal Permeability through CaCo-2 Cell Models. Antioxidants.

[46] André R., Pacheco R., Alves A.C., Santos H.M., Bourbon M., Serralheiro M.L. (2023). The Hypocholesterolemic Potential of the Edible Algae Fucus Vesiculosus: Proteomic and Quantitative PCR Analysis. Foods.

[47] André R., Guedes L., Melo R., Ascensão L., Pacheco R., Vaz P.D., Serralheiro M.L. (2020). Effect of Food Preparations on in Vitro Bioactivities and Chemical Components of Fucus Vesiculosus. Foods.

[48] Hurtado-Fernández E., Pacchiarotta T., Gómez-Romero M., Schoenmaker B., Derks R., Deelder A.M., Mayboroda O.A., Carrasco-Pancorbo A., Fernández-Gutiérrez A. (2011). Ultra High Performance Liquid Chromatography-Time of Flight Mass Spectrometry for Analysis of Avocado Fruit Metabolites: Method Evaluation and Applicability to the Analysis of Ripening Degrees. J. Chromatogr. A.

[49] Ford N.A., Spagnuolo P., Kraft J., Bauer E. (2023). Nutritional Composition of Hass Avocado Pulp. Foods.

[50] Waly D.A., Zeid A.H.A., Attia H.N., Ahmed K.A., El-Kashoury E.S.A., El Halawany A.M., Mohammed R.S. (2023). Comprehensive Phytochemical Characterization of Persea Americana Mill. Fruit via UPLC/HR-ESI–MS/MS and Anti-Arthritic Evaluation Using Adjuvant-Induced Arthritis Model. Inflammopharmacology.

[51] Gavicho Uarrota V., Fuentealba C., Hernández I., Defilippi-Bruzzone B., Meneses C., Campos-Vargas R., Lurie S., Hertog M., Carpentier S., Poblete-Echeverría C. (2019). Integration of Proteomics and Metabolomics Data of Early and Middle Season Hass Avocados under Heat Treatment. Food Chem..

[52] Ma W., Heianza Y., Huang T., Wang T., Sun D., Zheng Y., Hu F.B., Rexrode K.M., Manson J.A.E., Qi L. (2018). Dietary Glutamine, Glutamate and Mortality: Two Large Prospective Studies in US Men and Women. Int. J. Epidemiol..

[53] Huang J., Khatun P., Xiong Y., Liu B., Zhao Y., Lyu Q. (2023). Intakes of Folate, Vitamin B6, and Vitamin B12 and Cardiovascular Disease Risk: A National Population-Based Cross-Sectional Study. Front. Cardiovasc. Med..

[54] Ellinger S., Reusch A., Stehle P., Helfrich H.P. (2012). Epicatechin Ingested via Cocoa Products Reduces Blood Pressure in Humans: A Nonlinear Regression Model with a Bayesian Approach. Am. J. Clin. Nutr..

[55] Qi X., Liu H., Ren Y., Zhu Y., Wang Q., Zhang Y., Wu Y., Yuan L., Yan H., Liu M. (2023). Effects of Combined Binding of Chlorogenic Acid/Caffeic Acid and Gallic Acid to Trypsin on Their Synergistic Antioxidant Activity, Enzyme Activity and Stability. Food Chem. X.

[56] Velderrain-Rodríguez G.R., Quero J., Osada J., Martín-Belloso O., Rodríguez-Yoldi M.J. (2021). Phenolic-Rich Extracts from Avocado Fruit Residues as Functional Food Ingredients with Antioxidant and Antiproliferative Properties. Biomolecules.

[57] Brai B.I.C., Falode J.A., Adisa R.A., Odetola A.A. (2020). Effects of Aqueous Leaf Extract of Avocado (Persea Americana) on Total Cholesterol, Triacylglycerols, Protein and Haematological Parameters in CCl4-Intoxicated Rats. Clin. Phytosci..

[58] Uchenna U.E., Shori A.B., Baba A.S. (2017). Inclusion of Avocado (Persea Americana) Seeds in the Diet to Improve Carbohydrate and Lipid Metabolism in Rats. Rev. Argent. Endocrinol. Metab..

[59] Miñón-Hernández D., Dorantes-Alvarez L., Guzmán-Gerónimo R.I., Alvarado-Olivarez M., Herrera-Meza S., Santiago-Roque I., Gutiérrez-López G.F., Gómez-Patiño M.B., Arrieta-Baez D. (2021). Avocado Creole Peel Ameliorates Metabolic Alterations Caused by a High Sucrose Fat Diet in a Wistar Rats Model. Plant Foods Hum. Nutr..

