# Supplementary material for: Bioactive Properties of Persea americana Peel Extract and Their Role in Hypercholesterolemia Management and Cardiovascular Health
Source: Foods. 2025 Jul 16;14(14):2482. doi: 10.3390/foods14142482 (PMC12294314; doi:10.3390/foods14142482)
Supplement: Supplementary file 1 [file foods-14-02482-s001.zip › foods-3720568-supplementary.pdf]

Supplementary Material

Bioactive properties of *Persea americana* Peel Extract and Their Role in Hypercholesterolemia management and Cardiovascular Health

Laura M. Teixeira [1,2,3], Catarina P. Reis [3,4*] and Rita Pacheco [2,5*]

[1] Departamento de Química e Bioquímica, Faculdade de Ciências, Universidade de Lisboa, 1749-016 Lisboa, Portugal
[2] Centro de Química Estrutural, Institute of Molecular Sciences, Faculdade de Ciências, Universidade de Lisboa, 1749-016 Lisboa, Portugal
[3] Institute for Medicines (iMed.ULisboa), Faculdade de Farmácia, Universidade de Lisboa, 1649-003 Lisboa, Portugal
[4] Instituto de Biofísica e Engenharia Biomédica (IBEB), Faculdade de Ciências, Universidade de Lisboa, 1749-016 Lisboa, Portugal
[5] Departamento de Engenharia Química, Instituto Superior de Engenharia de Lisboa, 1959-007 Lisboa, Portugal
* Correspondence: R.P. rita.pacheco@isel.pt; C.P.R. catarinareis@ff.ulisboa.pt

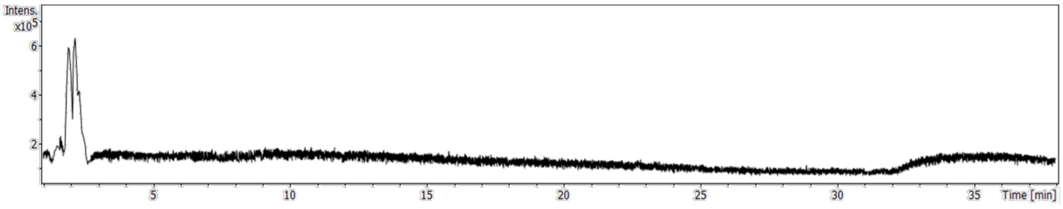

(a)

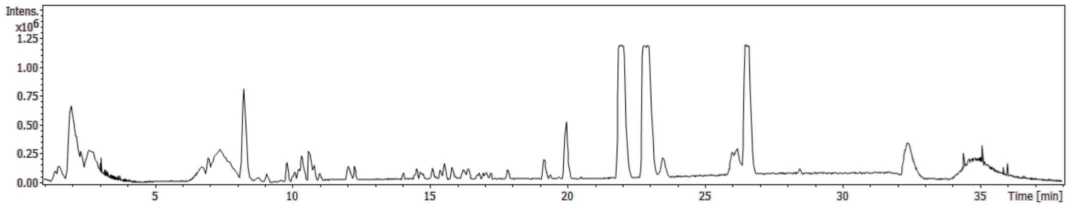

(b)

Figure S1. Chromatogram of the extract obtained through UHPLC-ESI-QTOF-MS/MS in the negative **(a)** and positive **(b)** ESI mode.

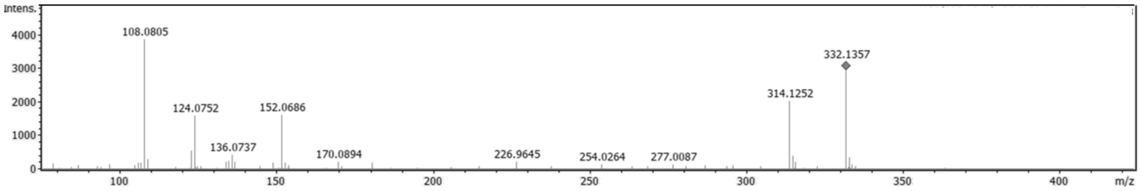

Figure S2. MS2 spectrum data for pyridoxine+ O-Hex **(6)**.
